# Supplementary material for: Strategies for developing sustainable health research capacity in low and middle-income countries: a prospective, qualitative study investigating the barriers and enablers to locally led clinical trial conduct in Ethiopia, Cameroon and Sri Lanka
Source: BMJ Open. 2017 Oct 13;7(10):e017246. doi: 10.1136/bmjopen-2017-017246 (PMC5652508; doi:10.1136/bmjopen-2017-017246)
Supplement: Supplementary material 1 [file bmjopen-2017-017246supp001.pdf]

Supplementary file 1: Comparison of barriers and enablers identified in the three case studies and the systematic review of health research capacity strengthening (Franzen, Chandler, Lang 2017)

Green tick indicates issues are the same, orange tick indicates issues are the same with minor exceptions where some issues are not mentioned or identified, red tick indicates some issues are the same but several points are not mentioned or identified. There were no contradictory findings.

General description of the barriers and enablers to locally-led trial undertaking, based on respondent reports in all three case studies	Comparison of findings between case studies and the systematic review of the literature			
	Ethiopia	Cameroon	Sri Lanka	Systematic Review (Franzen et al. 2017)
**Stewardship & governance**				
**Inefficient governance -** Largely bureaucratic, centralised hierarchies & strongly formalised organisational management structures leads to complex, multiplicative governance & permissions. This was often associated with administrative not research based leadership promotion, poor performance norms, competitive professional relationships, & resistance to streamlining, bottom-up initiatives, & delegating responsibility.	✓ (orange) Hierarchy not mentioned as problematic	✓ (green)	✓ (green)	✓ (orange) Problems rarely attributed to hierarchies
**Weak research stewardship -** Lack of strategy leads to supply-led, largely academic research & fragmented evidence of limited use for policy. Priorities may exist but limited local funding means agendas often foreign-led, sometimes inappropriately. Decision-makers may lack knowledge or appreciation for research due to administrative promotion. This de-values local research, prevents research cultures & can result in suspicion & blocking of research. Greater national investment & strategy required. Situation slowly improving due to local & foreign commitments.	✓ (green)	✓ (green)	✓ (orange) Research appreciated and strong research cultures in academia	✓ (green)
**Bureaucratic administration** introduces operational delays & permits low performance norms. Requirement for multiple permissions slows operations & encourages research "blocking". Financial regulations inhibit purchasing. Lack of research services, little appreciation for administration, & poor research-administrator engagement increase problems. This frequently results in researchers setting up parallel structures to bypass local systems. To overcome this, performance targets with clear accountability, institutional capacity development to manage research, & closer engagement needed.	✓ (orange) Administration problems reported but no solutions offered	✓ (green)	✓ (orange) Parallel structures rare as most research locally-led & institution-based	✓ (green)
**Weak regulatory frameworks** have limited review & monitoring capacity, are often overly complicated & cautious, & lack legal backing. This slows review times, limits scope of trials permitted & fuels ethical concerns. Poor quality applications also cause delay. More training in research ethics & trial design needed for reviewers & researchers. Committees need greater resources & legal backing. Increasing government commitment needed.	✓ (green)	✓ (green)	✓ (green)	✓ (green)

**Financing**				
**Research priority and finances for research -** Little top-level appreciation for research & evidence-based medicine. Universities prioritise teaching over research & research cultures often lacking. Investigators forced to apply for international funds but success is rare. This reduces the quantity & scope of research and increases dependence on foreign collaboration. To increase the value of research, advocacy of research benefits is needed. To gain international grants, skills in writing quality research proposals & international partnership are needed. Pilot research grants may support local studies.	✓	✓	✓ Low value national grants available. Research supported in academia. Little dependence on collaboration except for international grants.	✓
**Creating and sustaining resources**				
**Limited material capacity** particularly in laboratories; limits the scope of trials that can be attempted, may prevent collaborations & means samples may have to be analysed abroad. Basic services are also problematic. Few journal subscriptions & poor internet limit information & communication access. Resource constraints reduce motivation & self-efficacy. Greater institutional investment needed.	✓	✓	✓ Basic services & internet generally not problematic	✓
**Lack of human capacity to conduct research** generally more limiting than material resources; due to lack of skilled personnel but also inefficient use of expertise. Skills gaps blamed on little research training in education & work, few knowledge resources, few research opportunities & limited mentorship. Efficient use of human resources prevented by: limited time, few research careers, low motivation, poor research environment, intellectual isolation, limited teamwork & collaboration. This can lead to brain drain.	✓	✓ Material resources perceived as more limiting than human.	✓ Local researcher isolation not problematic. Brain drain not mentioned.	✓
**Developing human resource capacity is critical to increasing research conduct -** Knowledge & skill development modalities include: research modules in curricula, work-based training, trainer-of trainer programmes, e-learning, networking & knowledge sharing, & mentorship. This also inculcates research culture by increasing exposure, motivating personnel & increasing self-efficacy. Didactic training alone not normally sufficient to initiate trials.	✓	✓	✓	✓ Benefits for motivation and self-efficacy less mentioned
**Trial experience is the best learning & development strategy.** It gives exposure to trials & new methods, raises standards, & increases skills. Foreign-trial experience preferred for developing technical skills, knowledge sharing, & easier operations. But procedural nature & lack of inclusion & autonomy frustrates researchers. Locally-led trial experience normally better at developing leadership capacity due to opportunities for responsibility and challenging work because improves learning, self-efficacy & motivation. Embedding trials important for developing institutional capacity. Strong teamwork dynamics improves learning.	✓	✓	✓	✓ Responsibility, challenging work and teamwork rarely emphasised.
**Awareness of trials & exposure to research important** for thinking about research conduct, inculcating a research culture & securing stakeholder buy-in. This reduces suspicion of trials & increases the value of research. Exposure to trials & research is limited by minimal research training, little knowledge sharing & mentorship, limited access to knowledge resources & few trials conducted. Conducting & seeing research, sharing experiences through departmental events, teaching research, & mentorship can increase exposure.	✓	✓	✓ Exposure not needed for academics	✓

**Low motivation to conduct research** prevents interest in trials & effective use of expertise. Difficult operations, few incentives, little time, few research careers, poor research environment & expectation of barriers were disincentives. Career recognition & professional development was as important as financial incentives if research was linked to career progression. If not, salary incentives are normally a prerequisite. However, intrinsic incentives such as responsibility, recognition and challenging work sometimes off-set this.	✓ Responsibility & challenging work not mentioned	✓	✓ Better incentives for academic compared to healthcare staff	✓ Little attention to motivational factors especially responsibility and challenging work
**Producing and using research**				
**Difficult operations reduce trial conduct & usefulness for policy;** operations are similar for most trials but task difficulty varies depending on severity of barriers & enablers. Start-up stage normally most difficult. Expectation of barriers reduces motivation & self-efficacy. Leadership capabilities & collaboration & teamwork help cope with barriers, but resolution is dependent on system-wide development.	✓	✓	✓	✓
**Low uptake of research for policy.** Fragmented research, limited scope & supply-driven academic research reduce usefulness of trial evidence. Limited appreciation & understanding of research by decision-makers reduces evidence use. Little researcher-policy engagement & poor dissemination reduces research impact. This reduces perceived value of local research. Evidence-based guidelines often have little impact due to resistance or poor delivery. International evidence has more impact than local because of international backing, credibility & greater availability. Greater research-policy engagement & capacity building needed.	✓ Few evidence-based policies & research of questionable use, but little other detail mentioned	✓ Efforts to address this, especially research-policy engagement through platforms.	✓ Uptake depends on policy programme. Preference for international evidence not mentioned.	✓
**Self-efficacy to conduct trials is an important for trial undertaking & leadership -** Researchers frequently lack self-efficacy to lead studies even if they have extensive previous foreign-trial experience. Self-efficacy is reduced by: perceived complexity of trials, limited knowledge, little exposure to trials, lack of support, & lack of responsibility and openness to bottom-up initiatives. Self-efficacy increases through: training opportunities, trial experiences, mentorship and support, exposure to successful trials, responsibility & ability to make contributions.	✓	✓	✓ Self-efficacy not problematic for academics	✓ Rarely mentioned
**Local collaboration & teamwork important for enabling trials** by: pooling resources to reach a critical mass, improving relationships with stakeholders, building team morale, encouraging knowledge sharing, facilitating operations, & making research more useful for policy. However, local collaboration & teamwork are rare. They are prevented by limited networking & poor professional relationships & preference for foreign partners. Collaboration & teamwork are strengthened by: strategic networking & communication & team building skills.	✓ Teamwork and communication not mentioned	✓	✓	✓ Local collaboration not often mentioned
**International collaboration enables research -** Longer-term partnerships usually better because they have greater local inclusion & teamwork dynamics. Most international collaborations develop parallel structures which limit local institutional development. To ensure beneficial partnerships, strong local leadership is essential.	✓	✓	✓	✓
**Networking is important** for forging local & international collaborations, building professional relationships & teamwork, & engagement with stakeholders. International networking is more established than local networking due to preference for international partners. Networking is prevented by not having formal contacts, not being aware of expertise & poor professional relationships. Networking is improved by networking events, registries of expertise and online tools. Communication and team building skills can help forge relationships.	✓ Skills in forging relationships not mentioned	✓	✓ Local expertise generally well known	✓ Skills in forging relationships not mentioned
